# Long-COVID and Post-COVID Health Complications: An Up-to-Date Review on Clinical Conditions and Their Possible Molecular Mechanisms

**DOI:** 10.3390/v13040700

**Published:** 2021-04-18

**Authors:** Bruno Silva Andrade, Sérgio Siqueira, Wagner Rodrigues de Assis Soares, Fernanda de Souza Rangel, Naiane Oliveira Santos, Andria dos Santos Freitas, Priscila Ribeiro da Silveira, Sandeep Tiwari, Khalid J Alzahrani, Aristóteles Góes-Neto, Vasco Azevedo, Preetam Ghosh, Debmalya Barh

**Affiliations:** 1 Laboratório de Bioinformática e Química Computacional, Departamento de Ciências Biológicas, Universidade Estadual do Sudoeste da Bahia (UESB), Jequié, Bahia CEP 45206-190, Brazil; bandrade@uesb.edu.br (B.S.A.); sergio.siqueira@uesb.edu.br (S.S.); wrasoares@uesb.edu.br (W.R.d.A.S.); fernandarangelfarma26@gmail.com (F.d.S.R.); andria.sfreitas@gmail.com (A.d.S.F.); prirs91@hotmail.com (P.R.d.S.); 2 Departamento de Saúde II, Universidade Estadual do Sudoeste da Bahia (UESB), Jequié, Bahia CEP 45206-190, Brazil; 3 Programa de Pós-graduação em Genética e Biologia Molecular, Universidade Estadual de Santa Cruz, Ilhéus, Bahia CEP 45662-900, Brazil; naianeoliveira059@gmail.com; 4 Laboratório de Genética Celular e Molecular, Departamento de Biologia Geral, Instituto de Ciências Biológicas, Universidade Federal de Minas Gerais, Belo Horizonte, Minas Gerais CEP 31270-901, Brazil; sandip_sbtbi@yahoo.com (S.T.); vascoariston@gmail.com (V.A.); 5 Department of Clinical Laboratories Sciences, College of Applied Medical Sciences, Taif University, P.O. Box 11099, Taif 21944, Saudi Arabia; ak.jamaan@tu.edu.sa; 6 Laboratório de Biologia Molecular e Computacional de Fungos, Departamento de Microbiologia, Insti-tuto de Ciências Biológicas, Universidade Federal de Minas Gerais (UFMG), Belo Horizonte, Minas Gerais CEP 31270-901, Brazil; arigoesneto@gmail.com; 7 Department of Computer Science, Virginia Commonwealth University, Richmond, VA 23284, USA; preetam.ghosh@gmail.com; 8 Centre for Genomics and Applied Gene Technology, Institute of Integrative Omics and Applied Bio-technology (IIOAB), Nonakuri, Purba Medinipur, West Bengal 721172, India

**Keywords:** pathophysiology, adverse effects, systemic effects, COVID-19, molecular mechanism

## Abstract

The COVID-19 pandemic has infected millions worldwide, leaving a global burden for long-term care of COVID-19 survivors. It is thus imperative to study post-COVID (i.e., short-term) and long-COVID (i.e., long-term) effects, specifically as local and systemic pathophysiological outcomes of other coronavirus-related diseases (such as Middle East Respiratory Syndrome (MERS) and Severe Acute Respiratory Syndrome (SARS)) were well-cataloged. We conducted a comprehensive review of adverse post-COVID health outcomes and potential long-COVID effects. We observed that such adverse outcomes were not localized. Rather, they affected different human systems, including: (i) immune system (e.g., Guillain–Barré syndrome, rheumatoid arthritis, pediatric inflammatory multisystem syndromes such as Kawasaki disease), (ii) hematological system (vascular hemostasis, blood coagulation), (iii) pulmonary system (respiratory failure, pulmonary thromboembolism, pulmonary embolism, pneumonia, pulmonary vascular damage, pulmonary fibrosis), (iv) cardiovascular system (myocardial hypertrophy, coronary artery atherosclerosis, focal myocardial fibrosis, acute myocardial infarction, cardiac hypertrophy), (v) gastrointestinal, hepatic, and renal systems (diarrhea, nausea/vomiting, abdominal pain, anorexia, acid reflux, gastrointestinal hemorrhage, lack of appetite/constipation), (vi) skeletomuscular system (immune-mediated skin diseases, psoriasis, lupus), (vii) nervous system (loss of taste/smell/hearing, headaches, spasms, convulsions, confusion, visual impairment, nerve pain, dizziness, impaired consciousness, nausea/vomiting, hemiplegia, ataxia, stroke, cerebral hemorrhage), (viii) mental health (stress, depression and anxiety). We additionally hypothesized mechanisms of action by investigating possible molecular mechanisms associated with these disease outcomes/symptoms. Overall, the COVID-19 pathology is still characterized by cytokine storm that results to endothelial inflammation, microvascular thrombosis, and multiple organ failures.

## 1. Introduction

Severe acute respiratory syndromes are caused by a variety of coronaviruses, such as those in the SARS and MERS families. In early 2020, we reached the first SARS-CoV-2 peak of transmission and contamination, which led to a large number of fatalities worldwide [1,2,3]. The COVID-19 pandemic did not distinguish between different socioeconomic levels, sex or age groups [4,5]. Initially, efforts were focused on controlling the infectious process in order to prevent lung attack and respiratory insufficiency [6], since acute pulmonary and respiratory impairment was observed (similar to other SARS variants) [7]. In infected individuals, altered levels of oxygenation in arterial blood and the depletion of gas exchange caused severe deficiencies and losses of pulmonary capacitance, compromising the basic functions of life [6,8].

Since the start of the COVID-19 pandemic, an increasing number of studies have focused on rapid diagnosis, development, and redirection of new therapies. However, it was discovered that SARS-CoV-2 is more than just a respiratory syndrome [9,10,11]. High levels of endogenous chemical substances produced in response to the inflammation caused by this virus are capable of generating alterations and disturbances in target tissues. They even go beyond the barriers of protection of the innate tissue immunity, reaching the systemic level through hematogenous transmission [12]. Furthermore, during the development of the sepsis process, a high level of proinflammatory cytokines (IL-6, IL-1 and TNF-α) with pleiotropic abilities have been found to interact with their high-density receptors, immune cells, and vasculature [13]. These cytokines can stimulate a large number of processes involved with the activation of immune cells in response to changes in the vascular environment, promoting greater adhesion and blood procoagulation [14]. As a consequence, this signaling stimulates immunity cells involved in chronic inflammatory processes that can lead to pulmonary degeneration, pulmonary fibrosis, loss of function (with impaired oxygenation, culminating in a state of delayed hypoxia), hypoxemia, and anoxia (in more severe conditions), ultimately causing death [15]. Patients who have cardiopulmonary or metabolic comorbidities (e.g., diabetes mellitus), autoimmune diseases, or who are undergoing any treatment that might compromise their immunity (e.g., chemotherapy, radiotherapy, or corticotherapy) have a higher risk of death [15,16,17,18,19].

It has been verified that increases in complement level, coagulation and tissue factors, plasminogen activating factor I, and von Willebrand factor are responsible for modifying the hemostatic environment, and promoting thromboembolic issues [13,20]. These organic structures can occlude vessels locally, as they reach other tissues over a long distance—but not when they come off. As the lung and heart are closely interconnected, poor circulation can bring these chemical mediators close to immune cells and thromboembolisms that cause direct or indirect cardiac complications [12]. Additionally, the hyperinflammatory response in the arteries and venules can induce endotheliitis [21].

The overexpression of the human ACE2 receptor (hACE2-R) in various tissues (especially in the vessels) allows the virus to spread over the vascular system and reach the entire organism through its hematogenous involvement. Due to a vicious cycle—one which involves the production of chemical mediators produced by M1 macrophages, a reduction in vascular hACE2-R receptor density caused by viral endocytosis, and an increase in angiotensin II levels—this both vascular and inflammatory effects (Reynolds et al., 2020). Furthermore, this mechanism stimulates the production of more hACE2-R through positive feedback [22]. These repeatedly stimulated cycles favor the spread of infection and subsequent increase in angiotensin II levels which play an important role in the pathophysiological process of COVID-19 by generating increased vasoconstriction, inflammation, and fibrosis [23,24]. 

Toll-like receptors (TLRs) are a family of proteins that act as sensors, helping the immune system to distinguish between its own elements and foreign elements (Martínez et al., 2020). SARS-CoV (and presumably also SARS-CoV-2) interacts with TLRs in the host cell membrane and increases the expression of gene 88 of the primary response to myeloid differentiation (MyD88). MyD88 is responsible for activating the nuclear transcription factor kappa β (NF-kβ) and promoting an inflammatory cascade that increases lung damage [25].

In MERS-CoV infections, it was observed that an increase in the expression of the MyD88 gene was associated with higher mortality [26]. On the other hand, SARS-CoV infection in murine models showed that NF-kB inhibition resulted in reduced lung damage and an increase in survival rates. Thus, this mechanism seems to be of particular importance in coronavirus infections [27], and can be more aggressive in SARS-CoV-2 [12,22]. High expression levels of NF-kβ play an important role in the hyperinflammation process, stimulating cytokine-producing genes (IL-1 IL-2 IL-6 TNF-α), chemokines (IL-8 and oxanthin), cell adhesion proteins (ICAM, V-CAM-I and E-selectin), and inducible enzymes (nitric oxide synthase and cyclooxygenase II) [28]. The physiological effects of these substances can amplify the inflammatory process—sometimes exceeding the limit of innate organic defense barriers against exogenous agents and creating an endogenous environment of oxidative and inflammatory stress [29].

Many pathophysiological mechanisms have been described for SARS-CoV-2 (COVID-19), a virus which persists pandemically in 2021. In this work, we reported important updates on novel pathological processes of this viral disease in order to better understand their related mechanisms. This could help in seeking out new therapeutic alternatives. We conducted a comprehensive review on the possible mechanisms of action for the symptoms of associated pathologies with late- and post-COVID-19.

## 2. Methods

### Literature Search Selection Criteria

Recently, using a novel multiomics approach, we reported symptoms, comorbid conditions, and possible long-term complications of COVID-19 with above 90% precision [30]. We used these identified disease conditions and symptoms—along with several new key words—to search the literature database and retrieve specific associations and correlations. We used the Scopus Database, which allowed an improved and highly specific search, using terms in query filters, including titles, abstracts, keywords, years of publication, and types of research. Moreover, this platform remains one of the largest global indexing and summary databases and includes peer-reviewed scientific content [31,32]. For our search process, we used the following keywords in various combinations/strings: COVID-19, SARS-CoV-2, nCoV, 2019-n-cov, autoimmune disease, rheumatoid arthritis, toxic shock syndrome, thromboembolism, intravascular disseminated coagulation or or ICD, pulmonary embolism, postviral pulmonary fibrosis, pulmonary thromboembolism, cardiac arrhythmia, heart failure, gastrointestinal symptoms, gastrointestinal complications, orroetic olfactory dysfunction, dysfunction taste, stroke, Guillain-Barré syndrome or GBS, encephalitis, encephalopathy, ischemic stroke, intracerebral hemorrhage, cerebrovascular disease, pulmonary thromboembolism, cardiac arrhythmia, heart failure, gastrointestinal symptoms, encephalopathy, erythematous, rash, macular rash, papular rash, maculopapular rash, pseudo-chilblain lesions, vesicular lesions, livedo, necrosis, oral ulcers, blisters, herpetiform lesions, skin rash, post-traumatic stress disorder or PTSD, depression, anxiety disorder, insomnia, impaired attention, anxiety, impaired memory, mood, depression, depressive disorders, anxiety disorder, somatoform pain disorder, panic disorder, chronic fatigue, autism spectrum disorder, attention deficit disorder, attention deficit hyperactivity disorder, HRT, hyperactivity. The keywords were selected based on the review carried out by Leung et al. in 2020 [9], and all the terms used were based on the criterion that they were present in the titles, abstracts, and keywords of the documents. There was no specificity of language. The inclusion of references was based on documents published in the last six months of 2020 which displayed information on short- and long-term adverse complications associated with COVID-19.

## 3. Results

**Figure 1:**
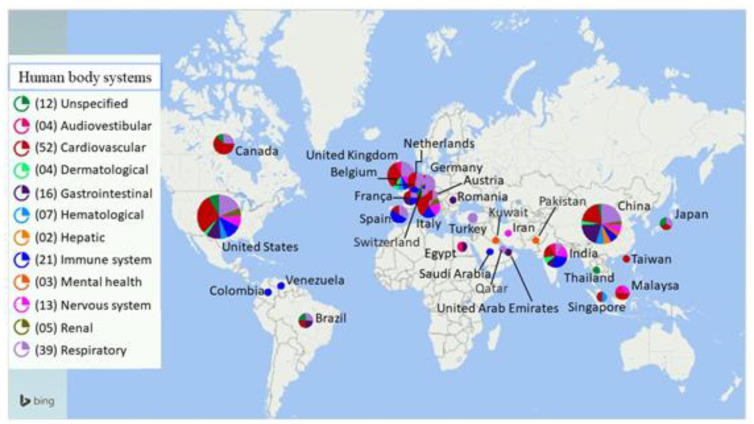
Geographical distribution of the studies used in this review and the number of articles reviewed per human body system. The map shows the countries where the articles were produced, as well as their respective approaches to human body systems affected by post- and long-term health complications associated with COVID-19.

The bibliographic search returned 2495 publications, but only 62 of this set of references were used to compose the results section. Furthermore, other relevant studies were added. In total, 134 publications–including systematic reviews, meta-analyses, case studies, clinical studies, and laboratory studies—were used as the bibliographic material for this review. Table 1 shows the number of articles according to keyword combinations. A geographical distribution of the articles used in the review is shown in Figure 1.

**Figure 2:**
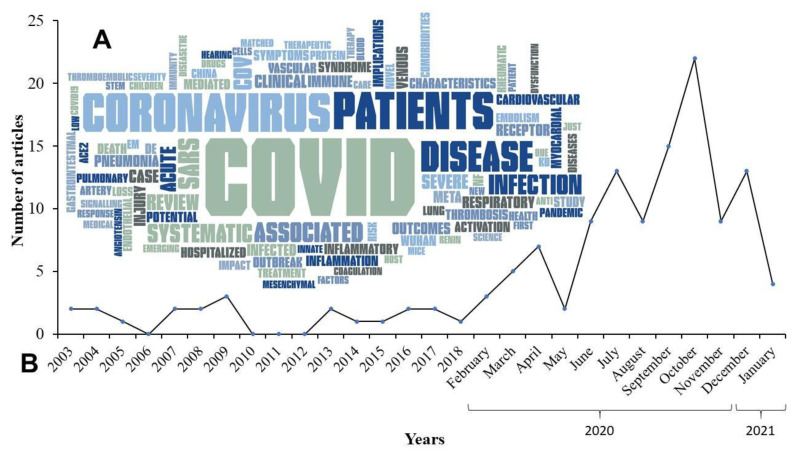
(**A**) Word cloud with the 100 most frequent words found in the titles of all studies that are part of this review, as well as a temporal analysis of the publications available in this review. (**B**) Frequency of publication (by year (2003-2018) and month (2020 to 2021)) of the 134 references used in this study.

Additionally, a word cloud reflects the most common terms related to this review study (Figure 2).

### 3.1. Complications of the Immune System

The chain of immunological events associated with SARS-CoV-2 is characterized by the evolution of adaptive immunity (mediated by T and B lymphocytes) to the virus [33,34].

Guillain-Barré Syndrome (GBS) has been associated to COVID-19. This disease is characterized by an abrupt evolution, with an inflammatory cascade of peripheral nerves and loss of the myelin sheath (polyneuropathy) [35]. GBS was reported in clinical studies of adult, young, and child patients during or after coronavirus infection [36,37]. Symptoms ranged from severe respiratory complications to motor paralysis. These symptoms have been associated—by different authors—to the physiological stimulation of inflammatory cells in COVID-19 infection. Nonetheless, further studies are required to determine how the COVID-19 virus induces this condition [35,38]. 

COVID-19-associated rheumatoid arthritis (RA) has been extensively detailed in case reports and observational studies [39,40]. An observational cohort study of COVID-19 by Pablos et al., 2020 [41] investigated preexisting inflammatory diseases in patients. The study identified 456 rheumatic patients with a mean age of 63 years, and demonstrated the highest risk factor for severe COVID-19 (28.1%) in positive patients in whom immunosuppressants were used continually. These results corroborated studies by Haberman et al. [42]. Additionally, a cross-sectional study of the impact of COVID-19 on rheumatic patients [42,43] observed that those affected exhibited arthralgia, myalgia, and weakness, with manifestations that preceded COVID-19 respiratory symptoms.

Autoinflammatory conditions were reported in children, including Kawasaki disease (KD) [44]. This disease predominantly affects children under five years old and is characterized as an acute inflammatory process in small and medium caliber vessels [45], exhibiting more cardiac involvement and a greater inflammatory response with macrophage activation [46]. Furthermore, myocarditis was found in young patients without any previous cardiac morbidity [46], with patients classified as critical, and with high cytokine secretion, showing acute respiratory distress syndrome. Despite the significant increase in KD cases after the start of the pandemic, further studies are needed to prove clinical association.

#### Molecular Mechanisms of Immune System Complications

A specific cytokine profile is associated with several factors in the severe stage of this disease, including: induction of interferon production, interleukin (ILs) 2 and 7 secretion, and the stimulation of granulocyte activation and production of tumor necrosis factor (TNF) [33,40], causing intravascular hyperinflammation with changes in angiogenesis and coagulation [41]. In addition to the understanding of the immune response to COVID-19, the association of symptoms with autoimmune diseases suggests that SARS-CoV-2 may trigger secondary diseases associated with a temporary immunosuppression profile and the presence of the virus [42,44]. 

### 3.2. Complications of the Hematological System

The pathophysiology of infection caused by COVID-19 involves several essential organic systems for maintaining homeostasis [12]. The direct effect of SARS-CoV-2 hyperinflammation induces the production of endogenous chemical substances that promote the alteration of vascular hemostasis [18]. Blood coagulation is directly affected by the release of procoagulant and proinflammatory cytokines [47] which activate disseminated intravascular coagulation and the formation of thromboembolic states that can aggressively affect various tissues, especially those that are more sensitive to ischemic processes, such as pulmonary, cardiovascular, and cerebrovascular tissues [13].

#### Molecular Mechanisms of Hematological System Complications

The SARS-Cov-2 cell entry mechanism is mediated by the hACE2-R receptor, which is expressed in several tissues, (e.g., lung, heart, intestinal smooth muscle, liver, and kidneys) as well as in immune cells and the vascular endothelium [18,24]. When the virus binds to the hACE2-R, it is sequentially internalized, leading to a decreased density of the receptor on the vascular tissue. This is associated with negative regulation of hACE2-R activity and accumulation of angiotensin II (Ang II), which causes vasoconstriction, profibrotic, and proinflammatory effects, as well as inflammation and tissue fibrosis [13]. The increased stimulation of inflammatory cytokines IL-1 and IL-6 by the activated M1 phenotype macrophages (Interferon-ɤ) and the excessive activity of Ang II bring endothelial activation, increased permeability, and coexpression of adhesion molecules, thus generating a prothrombotic phenotype [20,48]. Furthermore, this is verifiable via an increased production of other substances (e.g, plasminogen activator inhibitor factor I (PAI), tissue factor (TF), and von Willebrand factor (vWF)), which generate hemostatic changes that leave the endothelium inflamed, preadhesive, and prothrombotic. This denotes ongoing tissue damage [47,49], which causes endotheliitis, mediated by SARS-CoV-2 directly invading endothelial cells [21].

### 3.3. Complications of the Pulmonary System

The SARS-CoV-2 pathophysiology is complex, affecting several organs and systems; however, the cardiopulmonary system is severely affected [50]. The lungs—as target organs of the respiratory system—suffer from gradual functional failure, which can be verified by hypoxia and the pathological findings of minimal, noninvasive autopsies performed on patients [15,51]. A growing number of clinically studied cases of SARS-CoV-2 infection have demonstrated that this virus affects the pulmonary system, inducing severe respiratory failure [50,52], while also generating extrapulmonary clinical manifestations [51,53,54].

Dysfunctions involving the respiratory system are among the most aggressive events associated with exacerbated immune responses caused by viral infection [55]. The cytokine storm activates defense processes, stimulating biochemical pathways and leading to the production of tissue injury markers and the collapse of lung tissue [56,57,58]. Among the main associated pathologies, we can highlight: respiratory failure [58,59], pulmonary thromboembolism [5,50,60], pulmonary embolism [61,62], pneumonia [57,63], pulmonary vascular damage [64], and postviral pulmonary fibrosis [55,65]. Pulmonary vein thrombosis is rarely found, but can present together with dyspnea, cough, chest pain, and/or hemoptysis, causing systemic arterial embolism [64]. Systemic arterial embolism, in turn, is associated with venous and arterial thromboembolism pathogenesis in hypercoagulable states in patients with COVID-19 [15,64,66]. In a study, clinical and pathological findings indicated the presence of diffuse bilateral alveolar damage (DAD) in all the patients with SARS-CoV-2 [15,51]. Macroscopic and microscopic necropsy findings in patients who had severe COVID-19 infection demonstrated the presence of a major pulmonary pathology, described as a common complication, in patients with COVID-19. The complication overlapped with acute bronchopneumonia (secondary), which was present in 78.6% of the patients and could be considered the main cause of death. Another important finding, in addition to that main pathology, was the presence of thrombotic/thromboembolic vascular occlusions [15,50]. These hematological changes have been classified into five types of pulmonary thrombi: (I) capillary microthrombi; (II) thrombi partially organized in medium-sized pulmonary arteries with complete occlusion of the vessel; (III) thrombi not organized in medium-sized pulmonary arteries that do not completely fill the lumen of the vessel and probably represent thromboembolism instead of thrombosis; (IV) bone marrow emboli; and (V) septic pulmonary thromboemboli [5,15,51,67].

Pulmonary thrombi in medium-sized arteries were observed in 35.7% of patients, causing pulmonary infarction and/or pulmonary hemorrhage [5]. After the autopsy results of the patients with postviral infection, it was confirmed that COVID-19 is a disease with a systemic characteristic [15,51]. Because of a high involvement of the lungs in the infection, it increases the risk of cardiac and vascular complications, including acute myocardial injury and thrombotic/thromboembolic events that can affect other organs [15,58]. Despite the evidence of pathological changes to secondary acute bronchopneumonia, it has been described as one of the most common complications in patients with COVID-19 and may be the leading cause of death [57,63].

#### Molecular Mechanisms of Pulmonary System Complications

New evidence, involving analysis of coexpression data in 130,000 transcriptomes of human lung cells, revealed that there are three physiological systems directly involved in the pathogenesis of COVID-19: (I) the kinin-kalicrein system; (II) the renin-system angiotensin; (III) and the coagulation system coexpressed with hACE2-R in alveolar cells [58]. These systems are physiologically regulated by hACE2-R, and when SARS-CoV-2 infects the lungs, it induces a lethal triad in critically ill patients, associated with respiratory failure, acute cardiovascular failure, and coagulopathy. Furthermore, hACE2-R overexpression during the infection can deregulate these systems, causing acute inflammatory pulmonary edema (kinin-kalicrein system), cardiovascular instability (renin-angiotensin system) and thromboembolism (coagulation system) [58]. Other biochemical and immunopathogenic mechanisms of SARS-CoV-2 suggest that hyperinflammation, activated immune cells, and inflammatory mediators (cytokines and chemokines) contribute to alveolar endothelium damage, leading to apoptosis of alveolar cells type I (ATI) and degeneration of pulmonary type II alveolar cells (ATII). ATI and ATII form the monolayer of the alveolar epithelium [55]. Under normal conditions, ATII cells secrete surfactant, covering the entire epithelium lining to facilitate expansion of the alveolus. Both cell types are closely connected, with narrow junctions that control the transfer of ions and fluid through the epithelium [68]. The capillary endothelial cells are connected by intercellular junctions and control the influx of inflammatory cells and fluid into the interstitial space [69]. SARS-CoV-2 infects resident ATII cells and alveolar macrophages that express hACE2-R, activates cytokine and chemokine secretion and aggregates immune cells (neutrophils and monocytes) that produce toxic mediators, causing endothelial and epithelial lesions, leading to the death of the alveolar cells, fibrin deposition, and hyaline membrane formation [24,68]. It also increases the permeability of inflammatory cells to migration, the influx of red blood cells (RBCs) and blood capillary fluid, and the accumulation of inflammatory liquid (alveolar edema), which fills the air space and causes difficulty in breathing [55].

### 3.4. Complications of the Cardiovascular System

Pathophysiological findings in patients with SARS-CoV and MERS-CoV have shown that both are associated with myocardial injury, myocarditis, and heart failure [70,71]. On the other hand, the main mechanisms for myocardial injury are heterogeneous, mainly involving the cardiopulmonary and vascular system [15]. In these cases, the cardiovascular system is affected in several ways by severe infection in the acute respiratory tract caused by SARS-Cov-2. Myocardial injury is detected in 25% of hospitalized patients with COVID-19, and is associated with an increased risk of mortality [13]. The incidence of major cardiovascular events, such as acute myocardial infarction Type I and II, associated with SARS-Cov-2 infection significantly increases the risk of cardiac injury. This, in turn, leads to a negative prognosis [72]. Increasing clinical evidence and epidemiological findings have shown that patients’ cardiovascular comorbidities may be associated with an increased risk of death caused by COVID-19 [7,73]. Furthermore, macroscopic histopathological findings at necropsy of patients with COVID-19 showed evidence of chronic heart disease, including myocardial hypertrophy (92.9%), mild to severe coronary artery atherosclerosis (100%), and focal myocardial fibrosis (21.4%). Acute myocardial infarction was found to be a concomitant cause of death in 21.4% of patients, and significant cardiac hypertrophy was present in 7.1% of patients with ATTR-positive cardiac amyloidosis [15].

COVID-19 has at least five commonly accepted pathophysiological mechanisms which impact the myocardium: (a) The rupture of atherosclerotic plaque observed in type I myocardial infarction [21,74,75,76]; (b) the imbalance between myocardial oxygen supply and demand in type II myocardial infarction [21]. From this second type of infarction, four specific mechanisms related to COVID-19 that seem to be relevant can be described: fixed coronary atherosclerosis limiting myocardial perfusion, endothelial dysfunction within coronary microcirculation, severe systemic hypertension resulting from high circulating levels of Ang II, and intense arteriolar vasoconstriction and hypoxemia resulting from acute respiratory distress syndrome (ARDS) or pulmonary vascular thrombosis in situ; (c) generalized infection (sepsis); (d) lung injury; and (e) respiratory failure, related to severe physiological stress that may be associated with elevations in the biomarkers of tension and myocardial injury [77,78].

Adverse pathophysiological responses—in combination with other factors, such as age, sex, cardiovascular and metabolic comorbidities—increase the risk of cardiac injury, functional failure and death [10,21,79]. Additionally, the use of therapeutic protocols that require polypharmacy (mainly the use of antiviral drugs) can cause mitochondrial dysfunction and cardiotoxicity. Several classes of drugs present in the treatment protocols of COVID-19 can increase the risk of cardiac injury [10]. Patients with special clinical conditions, e.g., cancer treatment, have a high risk of chemotherapeutic cardiotoxicity associated with COVID-19 infection [19]. The intensification of cardiac injury can happen in the presence of other clinical conditions, such as arrhythmia, thrombosis, pericardial disease, myocarditis, heart failure and Takotsubo syndrome [80].

#### Molecular Mechanisms of Cardiovascular System Complications

Severe systemic inflammation in patients with SARS-CoV-2 is one of the causes of myocardial injury. High levels of circulating cytokines and mediators of toxic response have been described, including IL-6, TNF-α, nitric oxide, and activity modulation of the calcium channel, respectively [81,82]. It is believed that the action of these mediators may generate myocardial depression in systemic hyperinflammatory states, including sepsis [22,83,84,85]. Additionally, augmented direct viral invasion of the myocardium has been described, in which a number of viral particles enter into the cardiac fibers as the myocardium show increased expression of the hACE2-R that binds to the spike of SARS-CoV-2 [18,23,86,87].

### 3.5. Complications of Gastrointestinal, Hepatic, and Renal System

Inflammatory complications involving the digestive system are not uncommon in individuals affected by COVID-19 [88,89,90]. Clinical manifestations, such as diarrhea, nausea, vomiting, abdominal pain, anorexia [91], acid reflux [88], gastrointestinal hemorrhage [92], lack of appetite [90], and constipation [93] have been reported in epidemiological studies of patients affected by the novel coronavirus. These symptoms can occur during the early stages of the disease, known as the viral phase, or manifest as long-term adverse gastrointestinal effects [90]. Gastrointestinal symptoms have been associated with the immune system and changes in intestinal flora, and are related to existing comorbidities in patients affected by the virus [90]. Obesity, old age, diabetes, nutritional diet, and malnutrition are among the factors that can contribute to systemic inflammation and dysfunction of intestinal metabolites [94,95,96].

A meta-analysis conducted with thirty-one studies on the prevalence of gastrointestinal symptoms in 4682 patients showed that the most significant gastrointestinal symptoms resulting from COVID-19 include diarrhea and anorexia [97]. Moreover, in this study, it was observed that the patients admitted to intensive care units or presenting with high severity had a higher probability of abdominal pain and increased hepatic inflammatory markers (e.g., aminotransferase aspartate, alanine aminotransferase). Another study reported that, among gastrointestinal clinical manifestations, vomiting and diarrhea were more common, with a total incidence of 17.7% (95% Cl 13.9–21.5%) in pediatric patients [98]. This study also showed a rate of 85.8% (91/106) among cases that tested positive for fecal nucleic acid in children with COVID-19. Knowing that the feces of patients with COVID-19 can test positive for SARS-CoV-2 nucleic acids, possible transmission of the disease through the fecal-oral route should also be considered [90,99].

The hyperinflammatory changes caused by COVID-19 in the cardiopulmonary vasculature can induce prothrombotic states that compromise blood flow to other organs. Renal infarctions (caused mainly by thromboembolism) in patients with COVID-19 are considered rare. Ischemic events have a low incidence of around 0.1–1.4%, while splenic thrombosis has not been frequently described [100]. The data suggest that COVID-19 infections can cause macro and micro thromboembolic renal dysfunction and trigger a microvascular obstruction and infarction [101,102]. Idilman et. al. [102] found that a large number of patients with mild to moderate COVID-19 had perfusion deficits (PD) in their lungs and kidneys, which may be suggestive of the presence of systemic microangiopathy with microthrombosis.

#### Molecular Mechanisms of Gastrointestinal, Hepatic, and Renal System Complications

Gastrointestinal symptoms caused by SARS-Cov-2 occur when the infection is associated with the lung-intestine-brain axis, where the virus activates intestinal receptors, inducing inflammation in the tissues and causing a high viral load that induces gastrointestinal problems [94]. The infection causes disturbances and reduction of intestinal microorganism colonies, which can activate immune cells and provoke the release of pro-inflammatory cytokines, causing dysbiosis of the microbiome of the infected individual and inducing an inflammatory environment that can increase systemic inflammation [94,99,103].

### 3.6. Complications of Skeletomuscular System

Musculoskeletal symptoms may be associated with the incidence of central and peripheral neurological symptoms [104]. Studies show that the incidence of neurological disorders can affect the central nervous system (24.8%) and peripheral nervous system (8.9%)—and can also cause skeletal muscle injuries (10.7%) [104].

Viral infections caused by SARS-CoV-2 can generate immune-mediated skin diseases [105,106]. The use of immunosuppressants as a way of decreasing hyperinflammatory reactions (characterized by hyperactivation of macrophages and high levels of pro-inflammatory cytokines in COVID-19 [105]) were related to the indirect involvement of the dermis, with skin manifestations occurring independently of the stage or severity of the disease [106,107]. Additionally, immune-mediated conditions, such as psoriasis and lupus, are associated with an increased risk of viral infection [106]. These could include dermatomyositis—an inflammatory myopathy that affects the skin and other organs, generating weakness and rashes—which has been reported in patients infected by SARS-CoV-2 [108]. Other associated diseases include systemic lupus erythematosus (SLE), which is a chronic rheumatological condition found as a direct manifestation of COVID-19, as well as several clinical conditions associated with vasculitis in patients with SARS-CoV-2 [107].

As epithelial manifestations of COVID-19 appear as damage to the perifollicular levels and erythematous plaques caused by the response mediated by primary cells, they can appear after the first general symptoms of the disease—mainly in adult heterogeneous patients [105,108]. In addition, these patients can present rashes, hives, and acrocyanosis at all ages [108].

#### Molecular Mechanisms of Skeletomuscular System Complications

The molecular mechanisms involved with skeletomuscular system complications start from the modulation of the expression of neuromuscular inflammatory endogenous markers [104]. There is a complex interaction—mediated by the contraction of skeletal muscle, involving the immune system and adipose tissue—contributing to the control of health status and neutralization of viral infectious processes [109]. Skeletal muscle, especially after regular physical activity, is responsible for the production of myokines (cytokines derived from muscle) that signal a state of physiological muscle inflammation, which, in the presence of the SARS-CoV-2, can be significantly intensified [109]. Muscle-derived cytokines (myokines) production stimulates human hACE2-R overexpression and creates an infectious turnover cycle, related to viral invasion in the peripheral nervous system and skeletal muscle. [109,110] Myokine production also stimulates a prolonged muscular inflammatory environment, with high levels of IL-6 [109]. In physiological muscle stimulation through physical activity, this myokine would induce an anti-inflammatory environment, reducing the number of macrophages subtype 1 (M1—pro-inflammatory) and increasing the number of macrophages subtype 2 (M2—anti-inflammatory). The SARS-CoV-2 infection induces an opposite state, increasing the rate of proinflammatory macrophages and stimulating an increase in IL-1, TNF-α and Toll-like receptors (TLRs) [109]. An analysis of human skeletal muscular system expression patterns showed that several cell types express the TMPRSS2 receptor, including vascular cells, endothelial cells, smooth muscle cells, pericytes, muscle stem cells, macrophages, adaptive immune cells, and myonuclei. On the other hand, only smooth muscle cells and pericytes express hACE2-R [111].

Primary SARS-CoV-2 respiratory infection induces systemic inflammation (CXCL10, IFN-ɤ, IL-1B, IL-6, IL-8, IL-17, TNF-α) that can impact the musculoskeletal system through the expression of hACE2-R and TMPRSS2 genes in various types of musculoskeletal cells, allowing direct viral infection. However, the mechanism of musculoskeletal infection is not fully understood, and requires further study [111,112].

Epidemiological data from the SARS pandemic from 2002 to 2004 identified some major musculoskeletal symptoms, including generalized weakness, fatigue, atrophy of muscle fibers, extensive myalgia, and muscle dysfunction as common sequelae in patients with moderate and severe forms of this disease [111,113]. This suggests that SARS-CoV-2 infection led to deficits in muscle strength and endurance, probably due to the proinflammatory effects of viral infection and the deconditioning that occurs during the recovery period [111,112,114]. In experimental animal studies, a rapid 20% reduction in body mass was observed 4 days after SARS infection [115], while a study in humans noted the following: generalized atrophy of muscle fibers, with sporadic necrosis, focal infiltrations of muscle fibers and infiltration of immune cells, derangement of myofibrils and flow of disc Z, and neuronal demyelination. All of these factors contributed to muscle weakness and fatigue [116,117,118].

### 3.7. Complications of Nervous System

The short- and long-term impacts on the central nervous system from COVID-19 infection are not clear. A retrospective multicenter study by Mao et al., 2020 [119] was the first study to assess neurological manifestations in COVID-19. The study found that they were present in 36.4% of 214 patients. The most common manifestations were of the CNS (24.8%), followed by manifestations of the peripheral nervous system (8.9%). The most reported symptoms in COVID 19 were loss of taste, smell and hearing, headaches, spasms, convulsions, confusion, visual impairment, nerve pain, dizziness, impaired consciousness, nausea and vomiting, hemiplegia, ataxia, stroke, and cerebral hemorrhage [120,121,122,123].

Anosmia and ageusia are initial simple neurological manifestations in most of the patients with COVID 19 [120]. The sudden loss of taste and smell have been officially listed by the United States Center for Disease Control and Prevention (CDC) as symptoms of COVID-19 [123]. In a meta-analysis study, researchers identified changes in smell in 35.8% (and taste in 38.5%) of patients. These symptoms probably have a pathological basis related to neurotropic infection to the gustatory or olfactory systems [122,123].

Hearing loss has also been reported in patients infected with SARS-CoV-2. A review study by Almufarrij et al., 2020 [124] investigated the possibility of the vestibular system being affected by the new coronavirus, in the same way as the abuse of ototoxic drugs such as azithromycin and hydroxychloroquine can contribute to changes in this system [125]. Additionally, Saniasiaya et al., 2020 [122] reported that hearing loss may be caused by the ability of SARS-CoV-2 to deoxygenate erythrocytes, promoting a hypoxic state in the auditory center, which can lead to irreversible damage.

Nonspecific neurological symptoms have been reported in some studies, ranging from myalgia at 1.8–62.5%, headache from 0.6–70.3%, and dizziness from 0.6–21% [120]. Epileptic seizures have also been associated with COVID-19 [126]. Monti et al., 2020 [127] related a patient with no history of the disease and no comorbidities presenting epileptic seizures. In this investigation, anti-NMDA receptor antibodies were found in the cerebrospinal fluid, possibly characterizing anti-N-methyl-D-aspartate encephalitis (rNMDA), which is a neuropsychiatric syndrome caused by immune-mediated processes, exhibiting autoantibodies in the cerebrospinal fluid (CSF) [127]. Studies have also linked COVID-19 to atypical postpartum reversible encephalopathy syndrome, even in a normotensive patient, during pregnancy [128]. It was posited that a peripartum infection with COVID-19 could have triggered cerebral autoregulatory dysfunction, leading to the breakdown of the blood-brain barrier and subsequent vasogenic edema [128].

Stroke in COVID-19 is a rare complication that has been widely reported. Stroke, as a complication, is accompanied by poor prognosis, with a mortality rate of 46.7%. Its etiology is multifactorial, but it can be favored due to thromboembolic events characteristic of the disease [129,130]. In a systematic review, the frequency and clinical characteristics of stroke were assessed in 183 COVID-19 patients. It was shown that the risk of stroke was directly associated with advanced age and comorbidities [131]. Another study by de Frisullo et al., 2020 [132] described the case of a woman with SARS-Cov-2 infection who exhibited no flu symptoms but suddenly presented speech disorder and left hemiparesis. Brain magnetic resonance imaging showed two small acute cerebral infarctions in the right prerolandic cortex [132].

Ischemic stroke—involving anterior circulation with occlusion of large vessels—can occur. It happens mainly in the early stages of convalescence [129,130,133], but it can also appear later [129,130,133,134]. On the other hand, an atypical increase in cases of arterial venous sinus thrombosis (TSA) (from 7.7% to 28.0%) was reported in hospitalized patients with pneumonia and associated with COVID-19 [133]. TSA is usually classified as a rare subtype of stroke that affects women and young people, with an incidence of 2 to 5 cases per million people. It can lead to serious neurological complications, including significant visual deficits and death [133].

#### Molecular Mechanisms of Nervous System Complications

The pathophysiological mechanism responsible for causing various symptoms in COVID-19 is the ability of SARS-CoV-2 to induce, in severe cases, a cytokine storm that triggers the coagulation cascade, causing thrombotic complications such as disseminated intravascular coagulation (DIC) [135]. The most reported cerebrovascular diseases were ischemic stroke, hemorrhagic stroke, and cerebral venous thrombosis [120].

Rhea et al., 2020 [121] believe that the neurotrophic and neuroinvasive capacity of SARS-CoV-2 is one of the main pathophysiological mechanisms responsible for causing various symptoms. When the Spike (S1) protein of the new coronavirus binds to hACE2-R, it is recognized and captured by human cells. The brain has vast expression of this class of receptors and is possibly susceptible to infection—leading to the involvement of important regions of this organ [122]. The presence of SARS-CoV-2 mRNA (as well as the ease of S1 in crossing the blood-brain barrier) was noted in the cerebrospinal fluid of rats, penetrating 11 different regions of the brain [121].

### 3.8. Complications and Impacts on Mental Health

In addition to physical pathologies, the COVID-19 pandemic placed a mental health burden on the global population [136,137,138]. Quarantine and self-isolation were the main measures adopted to prevent the spread of the disease, resulting in an abrupt change in people’s lifestyles [137], bringing panic and anxiety to a significant number of individuals [136].

In a meta-analysis involving 62382 participants, in nineteen studies, stress was identified as the most prevalent mental health consequence (48.1%) of the COVID-19 pandemic, followed by depression (26.9%) and anxiety (21, 8%) [136,138]. In another study by Burhmah et al., 2020 [137] with more than 4000 people in Kuwait, they found that more than 50% of the interviewees had symptoms of depression and anxiety, and about 30% of them had relatives and friends with a diagnosis of COVID-19. In the same study, 39% were released from work, and over 37% spent more than 2 h a day following news about the pandemic [137].

Health professionals working to combat COVID-19 have been more severely affected by psychiatric disorders associated with depression, anxiety, insomnia, stress, and indirect trauma than other occupational groups [139]. In a meta-analysis with 10,267 health professionals from the pandemic front-line, depression was found in 31.5% in this class [136]. Most of the affected professionals were female (69.31%), married (59.37%), aged 21 to 30 years (23.84%) and nonsmokers (81.46%) [138].

The negative psychosocial effects of COVID-19 have been underestimated, and there is scant data available on the impact of this disease on mental health or the measures taken to limit its spread in the general population or in people who suffer from it [136].

### 3.9. Is the COVID-19 an Inflammatory Disease Related to Thrombosis?

Since the beginning of the COVID-19 pandemic, the severity of the disease has been associated with markers of coagulation disorders and independently associated with the development of respiratory failure, hypoxia and death [140]. In more severe cases, SARS-CoV-2 induces an intensified inflammatory process (cytokine storm) that ultimately results in the activation of the coagulation cascade, which causes various thrombotic phenomena, compromising adequate blood supply for many organs of the body [141]. Moreover, hemodynamic disorders in coagulation can affect sensitive organs with hypoxia, including the heart, brain, and lungs—compromising the maintenance of the patient’s physiological functions and potentially causing death [140].

The prothrombotic state seems to be the main contributing cause of diverse and devastating prognoses of severe COVID-19. This disease has been described to have association with hypercoagulable states and thromboembolic events in major blood vessels, the pulmonary artery, and major limb vessels. These lead to limb ischemia, associated with neurological symptoms, and complications, including stroke, macro and micro thromboembolic activities (which affect renal dysfunction) or infarction [12,142,143,144].

The activation of the innate immune system and the discharge of large amounts of the substances involved in the process of vascular inflammation also contribute to the aggressiveness of SARS-CoV-2 infection. The vicious cycle in the production of Angiotensin II (Ang II) and downregulation of hACE2-R is expressed in a massive way in the arterial and venous endothelium. This brings about a conducive environment that disseminates intravascular coagulation. Thus, the viral infection penetrates into the vascular endothelium, reducing the density of hACE2-R receptors and generating a chain of events (as a result of the inflammatory action of Ang II) that induces a proadhesive environment for aggregation and migration of macrophage inflammatory cells, leukocytes and lymphocytes. These produce interferon-gamma, TNF-alpha, IL-1, IL-6, and profibrotic factors such as tissue factor, plasminogen activating factor-1, and von Willebrand factor (Liu et al., 2020; Escher et al., 2020; Zachariah et al., 2020; Varga et al., 2020; Hamming et al., 2007; Boisrame-Helms et al., 2013). Because of these conditions, heparin and similar drugs have shown positive pharmacological effects on disseminated coagulation and vascular inflammation [145,146]. Furthermore, they may have other nonanticoagulant and anti-inflammatory effects, being relevant in the clinical aspects of the infected patients. Therefore, several scientific studies have indicated that these nonanticoagulant properties may be involved with the following immune-inflammatory mechanisms: (a) binding to inflammatory cytokines, (b) inhibition of neutrophil chemotaxis and leukocyte migration, (c) neutralization of the peptide complement factor positively charged C5a, and (d) sequestration of acute phase proteins [147,148,149,150]. Thus, heparin may decrease the levels of inflammatory biomarkers. However, in order to prove these indications, further studies must be carried out [151].

Alternative drug treatments in experimental drug repositioning protocols can help reduce the aggressive effects of hyperinflammation generated by COVID-19 [152]. The statin drugs are used to treat coronary heart disease, and have biochemical mechanisms related to the inhibition of proinflammatory routes, as well as direct inhibition of the main protease of the virus [152]. Their most common alteration in pharmacokinetic profiles is through the elevation of aminotransferases (AST and ALT), with occasional increases in alkaline phosphatase and total bilirubin [153]. Although there is little scientific evidence about the pharmacodynamic and toxicological aspects of these drugs [153,154], it has been shown that the use of statins in patients with COVID-19 is safe [152].

COVID-19 commonly involves the cardiovascular system [54,155]. Between 1/5 and 1/3 of studied hospitalized patients displayed evidence of myocardial injury—defined as the presence of high levels of biochemical markers upon admission. Cardiac troponin (hsTnI) is the gold standard of reference for the diagnosis of myocardial injury [16,156]. These patients were generally elderly and had a higher prevalence of hypertension, diabetes mellitus, coronary artery disease, and heart failure than those with normal troponin levels [56,157].

Myocardial injury is associated with a greater need for mechanical ventilatory support and higher in-hospital mortality [13]. Although the heart is a central and important organ for the maintenance of body homeostasis, several other important organs are also involved, and the direct or indirect impact of the virus on the myocardium can affect the maintenance of blood pressure levels, blood clotting and, consequently, tissue oxygenation patterns [66].

The previously-described symptoms are among the main manifestations observed in the lung tissue that can lead to the acute failure of the cardiorespiratory system of patients with severe COVID-19 [56]. Although it was originally believed to be a syndrome characterized by acute lung injury, respiratory failure and death, it now appears that this pathology is characterized by an exuberant cytokinemia, with resulting endothelial inflammation, microvascular thrombosis, and multiple organ failure [2].

## 4. Conclusions and Perspectives

**Figure 3:**
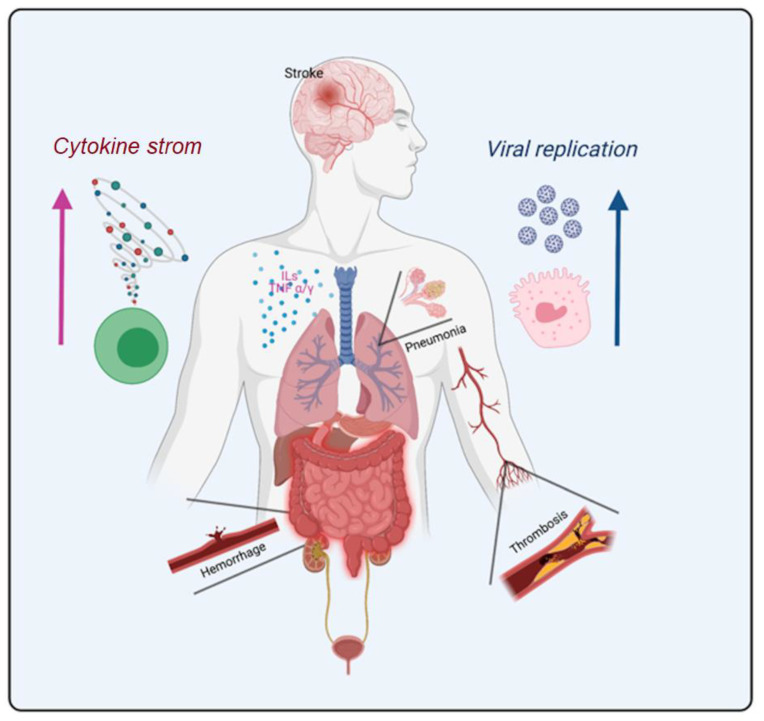
The most relevant human body systems affected by the long-COVID-19 and post-COVID-19 associated symptoms found in articles used in this review.

There is a commonality to all the symptomatology and systemic pathophysiological changes presented so far for COVID-19: they always involve organic systems integrated with hematological and vascular dynamics. The main human body systems affected by the long- and post-COVID-19 symptoms are represented in Figure 3.

Viral infection leads to an aggressive immunological reaction, which directly and indirectly compromises the cardiopulmonary system. However, hematological changes caused by vascular inflammation bring about a microenvironment for thromboembolism formation, affecting other vital organs, such as the nervous system, gastrointestinal tract, liver, and kidneys. In addition, this viral infection can lead to destabilization of the liver, which signals a state of maximum alertness for the whole organism, and generates an increase in plasma biochemical markers of tissue aggression produced in response to tissue-damaging agents. Table 2 summarizes all post- and long-term COVID-19 symptoms for each human body system described in this review [6,9,34,50,56,96,99,118,121,122].

Prospects for the evolution of COVID-19 systemic pathology are aimed at assessing its long-term degenerative effects and sequelae, as presented in perennial (myocardium and brain) and stable (soft tissue) organs. Furthermore, late viral infection has been related to other disorders, pathophysiological changes in pregnant women and fetuses, memory and reasoning profile alterations, muscle and joint pain, and alteration of the cardiac QT wave interval. Some of these may be involved in sensitizing the immune system to viral particles such that, even though they are eliminated from the organisms, they maintain a latent immune memory. This is currently one of the main hypotheses proposed to explain the late-COVID-19 effects in some organs. Even in the more protected tissues, viral particles can be found due to their ability to invade the vascular tissue (arteries and veins).

Adverse postvaccination responses may be a source of study in the coming months. Moreover, new therapies involving reproposition of drugs applied to vascular inflammation and anticoagulation, new molecular targets involved in the dynamics of the pathogenesis of COVID-19 for the development of chemical inhibitors, and cell therapies with mesenchymal stem cells (MSCs) also deserve to be seriously investigated.

## Figures and Tables

**Table 1:** Detailed bibliographic search results found in the Scopus database.

^1^ Keywords/Strings	^2^ Number of Recovered Articles
TITLE-ABS-KEY (Covid-19 OR sars-cov-2 OR 2019-n-cov AND “autoimmune disease” OR “rheumatoid arthritis” OR “toxic shock syndrome” OR thromboembolism) AND (LIMIT-TO (DOCTYPE, “ar”) OR LIMIT-TO (DOCTYPE, “re”))	973
TITLE-ABS-KEY (Covid-19 OR sars-cov-2 OR 2019-n-cov AND “intravascular disseminated coagulation” OR ICD OR “pulmonary embolism” OR “Postviral pulmonary fibrosis” OR “pulmonary thromboembolism” OR “cardiac arrhythmia” OR “heart failure” OR “gastrointestinal symptoms” OR “gastrointestinal complications” OR “Orroetic olfactory dysfunction” OR ortorium “Dysfunction taste” OR stroke OR stroke OR GBS OR encephalitis OR encephalopathy OR “ischemic stroke” OR “intracerebral haemorrhage” OR “cerebrovascular disease”)	125
TITLE-ABS-KEY (Covid-19 OR sars-cov-2 OR 2019-n-cov AND “pulmonary thromboembolism” OR “cardiac arrhythmia” OR “heart failure” OR “gastrointestinal symptoms” OR encephalopathy) AND (LIMIT-TO (DOCTYPE, “ar”) OR LIMIT-TO (DOCTYPE, “re”))	1398
TITLE-ABS-KEY (Covid-19 OR sars-cov-2 OR 2019-n-cov AND ‘erythematous AND rash’ OR “macular rash” OR “papular rash” OR “maculopapular rash “ OR “ pseudo-chilblain lesions “ OR “ vesicular lesions “ OR livedo OR necrosis OR “ oral ulcers “ OR blisters OR “ herpetiform lesions “ OR “skin rash” OR “post-traumatic stress disorder “ OR ptsd OR depression OR “ anxiety disorder “)	20
TITLE-ABS-KEY (Covid-19 OR sars-cov-2 OR 2019-n-cov AND insomnia OR “impaired attention” OR anxiety OR “impaired memory” OR mood OR depression OR “depressive disorders” OR “anxiety disorder” “somatoform pain disorder” OR “panic disorder” OR “Chronic fatigue” OR “autism spectrum disorder” OR “attention deficit disorder” OR HRT OR hyperactivity)	65

^1^ Keywords used in searches in the Scopus database; ^2^ Number of retrieved publications for each keyword combination submitted to the Scopus database.

**Table 2:** Classified long-COVID and Post-COVID symptoms summarized as per human body system.

System	Symptoms	Post-COVID	Long-COVID
**Audio vestibular**	Ageusia	**X**	
	Anosmia	**X**	
	Hyposmia	**X**	
	Hearing Loss	**X**	
**Cardiovascular**	Myocarditis	**X**	
	Heart Failure	**X**	
	Myocardial Hypertrophy	**X**	
	Mild to severe coronary artery atherosclerosis	**X**	**X**
	Focal myocardial fibrosis		**X**
	Acute myocardial infarction Type I		**X**
	Acute myocardial infarction Type II		**X**
	Cardiogenic shock	**X**	
	Arrhythmia	**X**	
	Pericardial disease	**X**	
	Takotsubo syndrome		**X**
	Chronic heart disease		**X**
	Severe coronary artery		**X**
**Dermatological**	Psoriasis		**X**
	Systemic Lupus Erythematosus		**X**
	Vasculitis		**X**
	Dermatomyositis		**X**
	Chronic rheumatological disease		**X**
**Gastrointestinal**	Diarrhea	**X**	
	Nausea	**X**	
	Vomit	**X**	
	Abdominal pain	**X**	
	Anorexia	**X**	
	Acid reflux	**X**	
	Gastrointestinal bleeding	**X**	
	Lack of appetite	**X**	
	Constipation	**X**	
	Changes in the lung-intestine-brain axis		**X**
	Changes in the intestinal flora		**X**
	Disorders and disintegration of intestinal microorganisms		**X**
	Microbiota dysbiosis		**X**
	Dysfunction of intestinal metabolites		**X**
**Hematological**	Breakdown of hemostasis	**X**	
	Endoteliitis	**X**	
	Disseminated intravascular coagulation	**X**	
	Prothrombotic phenotype	**X**	
	Coagulative disease	**X**	
**Hepatic**	Alteration of inflammatory biomarkers of liver damage	**X**	
	Macro and micro thromboembolic	**X**	
**Immune system**	Secondary autoimmune symptoms associated with immunosuppression	**X**	
	Vascular inflammation and myocarditis	**X**	
	Guillain-Barret syndrome	**X**	
	Motor paralysis	**X**	
	Rheumatoid arthritis	**X**	
	Arthralgia	**X**	
	Myalgia	**X**	
	Weakness	**X**	
	Kawazaki disease	**X**	
**Mental Health**	Depression	**X**	
	Panic syndrome	**X**	
	Anxiety	**X**	
	Stress	**X**	
	Psychiatric disorders	**X**	
	Anguish	**X**	
	Insomnia	**X**	
	Negative psychosocial effects	**X**	
	Panic Syndrome	**X**	
**Nervous system**	Headaches	**X**	
	Spasms	**X**	
	Convulsions	**X**	
	Confusion	**X**	
	Visual impairment	**X**	
	Nerve pain	**X**	
	Dizziness	**X**	
	Conscience problems	**X**	
	Nausea	**X**	
	Vomiting	**X**	
	Hemiplegia	**X**	
	Ataxia	**X**	
	Stroke (AVC)	**X**	
	Cerebral hemorrhage	**X**	
	Nonspecific neurological symptoms		
	Epileptic seizures		**X**
	Myalgia		**X**
	Anti-N-Methyl-D-Aspartate encephalitis (rNMDA)		**X**
	Atypical postpartum reversible encephalopathy syndrome	**X**	
**Renal**	Renal disfunction	**X**	
	Renal systemic microangiopathy with micro-thrombosis	**X**	
**Pulmonary**	Pulmonary infarction	**X**	
	Pulmonary Hemorrhage	**X**	
	Respiratory failure	**X**	
	Pulmonary thromboembolism	**X**	
	Pulmonary embolism	**X**	
	Pneumonia	**X**	
	Secondary bronchopneumonia	**X**	**X**
	Pulmonary vein thrombosis	**X**	
	Post-viral pulmonary fibrosis	**X**	**X**
	Chronic respiratory failure	**X**	**X**
	Dyspnea	**X**	
	Cough	**X**	
	Chest pain	**X**	
	Hemptysis	**X**	
**Skeletomuscular**	Dermatomyositis		**X**
	Generalized weakness		**X**
	Fatigue		**X**
	Muscle fiber atrophy		**X**
	Extensive myalgia		**X**
	Muscle dysfunction		**X**
	Deficit in muscle strength and endurance		**X**
	Generalized muscle atrophy		**X**
	Sporadic and focal necrosis of muscle fibers		**X**
	Neuronal demyelination		**X**

## Data Availability

Not applicable.
